# Identifying hub genes of sepsis-associated and hepatic encephalopathies based on bioinformatic analysis—focus on the two common encephalopathies of septic cirrhotic patients in ICU

**DOI:** 10.1186/s12920-023-01774-7

**Published:** 2024-01-11

**Authors:** Juan Li, Dong Yang, Shengmei Ge, Lixia Liu, Yan Huo, Zhenjie Hu

**Affiliations:** 1 Department of Intensive Care Unit, Hebei Key Laboratory of Critical Disease Mechanism and Intervention, The Fourth Hospital of Hebei Medical University, Shijiazhuang, 050011 Hebei China; 2 Department of Emergency (Xiangjiang Hospital), The Third Hospital of Hebei Medical University, Shijiazhuang, 050051 Hebei China

**Keywords:** ICU, Septic cirrhotic patients, Sepsis-associated encephalopathy, Hepatic encephalopathy, Bioinformatic analysis, Autopsy human brain tissue

## Abstract

**Background:**

In the ICU ward, septic cirrhotic patients are susceptible to suffering from sepsis-associated encephalopathy and/or hepatic encephalopathy, which are two common neurological complications in such patients. However, the mutual pathogenesis between sepsis-associated and hepatic encephalopathies remains unclear. We aimed to identify the mutual hub genes, explore effective diagnostic biomarkers and therapeutic targets for the two common encephalopathies and provide novel, promising insights into the clinical management of such septic cirrhotic patients.

**Methods:**

The precious human post-mortem cerebral tissues were deprived of the GSE135838, GSE57193, and GSE41919 datasets, downloaded from the Gene Expression Omnibus database. Furthermore, we identified differentially expressed genes and screened hub genes with weighted gene co-expression network analysis. The hub genes were then subjected to Gene Ontology and Kyoto Encyclopedia of Genes and Genomes pathway functional enrichment analyses, and protein-protein interaction networks were constructed. Receiver operating characteristic curves and correlation analyses were set up for the hub genes. Finally, we explored principal and common signaling pathways by using Gene Set Enrichment Analysis and the association between the hub genes and immune cell subtype distribution by using CIBERSORT algorithm.

**Results:**

We identified seven hub genes—*GPR4*, *SOCS3*, *BAG3*, *ZFP36*, *CDKN1A*, *ADAMTS9*, and *GADD45B*—by using differentially expressed gene analysis and weighted gene co-expression network analysis method. The AUCs of these genes were all greater than 0.7 in the receiver operating characteristic curves analysis. The Gene Set Enrichment Analysis results demonstrated that mutual signaling pathways were mainly enriched in hypoxia and inflammatory response. CIBERSORT indicated that these seven hub genes were closely related to innate and adaptive immune cells.

**Conclusions:**

We identified seven hub genes with promising diagnostic value and therapeutic targets in septic cirrhotic patients with sepsis-associated encephalopathy and/or hepatic encephalopathy. Hypoxia, inflammatory, and immunoreaction responses may share the common downstream pathways of the two common encephalopathies, for which earlier recognition and timely intervention are crucial for management of such septic cirrhotic patients in ICU.

**Supplementary Information:**

The online version contains supplementary material available at 10.1186/s12920-023-01774-7.

## Introduction

Sepsis—a major global healthcare concern—is one of the leading causes of morbidity and mortality in intensive care units (ICUs) [1, 2]. Cirrhosis is an independent risk factor for sepsis and sepsis-related mortality [3]. In ICU wards, cirrhotic patients are more susceptible to sepsis and severe complications than the general population because of their easier intestinal microbiota translocation, systemic immune dysregulation, and hyperdynamic circulation with high cardiac output and low systemic vascular resistance [4–9].

According to statistics, 30% of hospitalized patients with liver cirrhosis have a bacterial infection. If they develop sepsis, their mortality rate will reach 40–90% [10, 11].

Sepsis-associated encephalopathy (SAE) and hepatic encephalopathy (HE) are two common neurological complications in septic cirrhotic patients. HE is a severe reversible metabolic complication of sepsis-induced liver cirrhosis, demonstrating clinical manifestations of minor cognitive dysfunction such as personality changes, memory decline, attention deficit, and other neurological changes, including lethargy, delirium, and coma [12, 13]. ICU admission is usually required when patients with severe sepsis frequently develop multiple organ dysfunction syndrome (MODS), such as acute respiratory distress syndrome, acute kidney injury, and SAE [14–17]. Up to 70% of patients may develop SAE with increasing severity of disease, which is a diffuse brain dysfunction without direct intracranial infection, and whose clinical manifestations are extremely similar to those of HE, ranging from cognitive dysfunction to neurological changes [18, 19].

The pathogenesis of both encephalopathies, SAE and HE, is complex. Some authors have suggested that SAE may have similar metabolic pathogenesis to that of HE based on clinical similarities, including a wide range of neuropsychiatric alterations, ranging from confusion and delirium to coma [20, 21].

Few studies have reported on the common underling molecule mechanisms. Mizock et al. found elevated levels of phenylacetic acid in the blood and cerebrospinal fluid in patients with SAE and HE, suggesting that phenylethylamine metabolites may contribute to encephalopathy in systemic sepsis and hepatic failure [21]. Sari et al. demonstrated that hyperventilation decreases cerebral blood flow and CBF/CMRO_2_ (cerebral blood flow/cerebral metabolic rate for oxygen consumption), indicating that purposeful or unconscious hyperventilation may produce cerebral ischemia in the respiratory management of SAE or HE patients [22]. However, bioinformatic analysis of reciprocal genes in SAE and HE has never been exhaustively explored until now.

We speculated that SAE and HE—the two kinds of encephalopathy—might share a mutual pathophysiological molecule mechanism and cross-talk genes. Herein, the present study explored the common hub genes and potential biological mechanisms using a bioinformatics approach with precious autopsy brain tissue in SAE and HE for identification and treatment, thereby providing new promising insights into the clinical management of such septic cirrhotic illnesses.

## Materials and methods

### Data source

To explore the mutual hub genes between SAE and HE of septic cirrhotic patients, we selected three independent cohorts (GSE135838 [23], GSE57193 [24, 25], GSE41919 [24]) from the Gene Expression Omnibus database (GEO), a global public bioinformatic repository comprising high-throughput microarray and next-generation sequence functional genomic datasets [26]. The RNA-seq data of GSE135838 was generated from 24 post-mortem human brain tissue samples from 12 sepsis cases and 12 non-sepsis controls (These cases were deprived of the Internal Medicine Division of Pulmonary and Critical Care Medicine, University of Michigan, USA) (https://www.ncbi.nlm.nih.gov/geo/query/acc.cgi?acc=GSE135838). The GSE57193 dataset comprises 12 post-mortem human brain tissue samples divided into 3 sample groups: 4 liver cirrhosis cases, 4 liver cirrhosis with HE cases, and 4 healthy controls (These cases were deprived of the Clinic for Gastroenterology, Hepatology and Infectiology, Germany) (https://www.ncbi.nlm.nih.gov/geo/query/acc.cgi?acc=GSE57193). The GSE41919 cohort involves 19 post-mortem human brain samples from 8 cirrhotic patients with or 3 without HE and 8 non-cirrhotic controls (These cases were deprived of the Clinic for Gastroenterology, Hepatology and Infectiology, Germany). (https://www.ncbi.nlm.nih.gov/geo/query/acc.cgi?acc=GSE41919). All cohorts were used for microarray expression analysis.

### Data extraction and management

All clinical, molecular information, and microarray datasets of these patients were publicly accessible at the GEO, and we used statistical analysis to investigate significantly differentially expressed genes (DEGs) on each microarray dataset. All aspects of experiment design, quality control, and data normalization conformed to standard Affymetrix protocols. The research was conducted in accordance with the International Conference and the Declaration of Helsinki.

### Statistical analysis

Each dataset was first evaluated for normality of distribution by using the Kolmogorov-Smirnov test to decide whether a non-parametric rank-based or parametric analysis should be performed. The Fisher exact and Wilcoxon rank-sum tests were used for hypothesis testing with categorical and continuous variables, respectively. DEGs expression analysis was performed using the limma package [27]. *P*-value < 0.05 in unpaired t-test analysis and fold change (logFC) > 0.5 or < − 0.5 were used to determine the DEGs. The core modules and hub genes related to SAE and HE were identified using a weighted gene co-expression network analysis (WGCNA) [28]. An appropriate soft-thresholding power β = 8 was selected by using the integrated function (pick soft threshold) in the WGCNA package. Constructing a weighted gene network entails the choice of the soft thresholding power to which co-expression similarity is raised to calculate adjacency. We also used CIBERSORT [29, 30] and Gene Set Enrichment Analysis (GSEA) [31] of gene expression profiles to identify immune cell infiltration characteristics and related core genes sets. The Clusterprofiler [32] package was used to identify Gene Ontology (GO) enrichment terms and Kyoto Encyclopedia of Genes and Genomes (KEGG) pathways [33–35]. For all statistical analyses, a *P*-value < 0.05 was considered significant. The numerical variables and categorical variables were compared using the Wilcoxon rank-sum, Kruskal-Wallis, and Fisher exact tests. Co-expression analysis was conducted to detect correlated expression levels among hub genes by calculating Pearson’s correlation coefficient. The confidence interval was 95%. All statistical analyses were performed using R software 4.1.0 [36, 37].

## Results

### Identification of DEGs and screening of hub genes

**Fig. 1:**
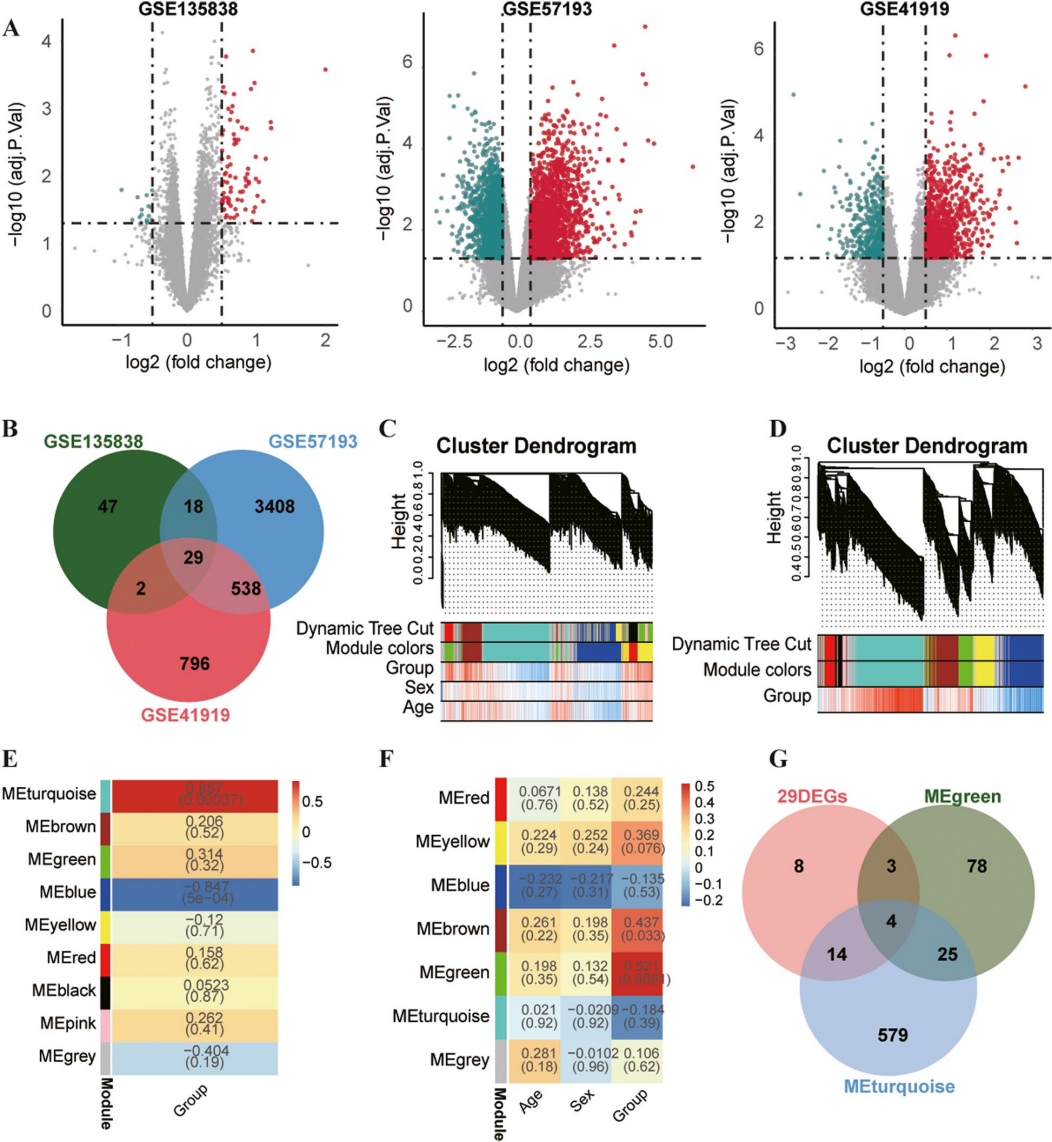
Identification of DEGs and screening of hub genes. **A** The volcanic plots of DEGs analysis in GSE135838, GSE57193, and GSE41919 datasets. **B** The Venn diagram shows 29 DEGs at the intersection of three datasets. **C**, **D** Cluster dendrograms. In terms of dynamic tree cut, the genes from GSE135838 and GSE57193 were clustered into different modules through hierarchical clustering. Diverse colors indicate respective different modules. **E**, **F** The associations between clinical traits and modules, in which red represents a higher correlation coefficient. GSE57193 involves a Turquoise module containing 622 genes (r = 0.857, *P*<0.001), whereas GSE135838 involves a Green module containing 110 genes (r = 0.521, *P*<0.01). **G** The Venn diagram shows the seven hub genes that overlap 29 DEGs, Turquoise module genes, and Green module genes

Based on the dataset GSE135838 (https://www.ncbi.nlm.nih.gov/geo/query/acc.cgi?acc=GSE135838), we observed 96 DEGs between 12 sepsis samples and 12 non-sepsis samples, comprising 80 up-regulated DEGs and 16 down-regulated DEGs (Fig. 1A). The data were screened in another dataset GSE57193 (https://www.ncbi.nlm.nih.gov/geo/query/acc.cgi?acc=GSE57193). There were 3993 DEGs between 4 liver cirrhosis patients with HE and 4 health controls, involving 2134 up-regulated DEGs and 1859 down-regulated DEGs (Fig. 1A). The third dataset was derived of GSE41919 (https://www.ncbi.nlm.nih.gov/geo/query/acc.cgi?acc=GSE41919), which shows 1365 DEGs between 8 liver cirrhosis patients with HE and 8 non-cirrhotic controls, including 912 up-regulated DEGs and 453 down-regulated DEGs (Fig. 1A). The intersection of 29 DEGs from the aforementioned three datasets was determined using a Venn diagram (Fig. 1B). We further screened clusters or modules of highly correlated genes by using WGCNA analysis, which applies to small sample sizes. The results included Green module 110 genes and Turquoise module 622 genes (Fig. 1C-F); the gene list for these two modules are presented in Additional file 1: Table S1. Through the overlapping of the 29 DEGs, Green module genes, and Turquoise module genes, we obtained the following seven hub genes: *GPR4*, *SOCS3*, *BAG3*, *ZFP36*, *CDKN1A*, *ADAMTS9*, and *GADD45B* (Fig. 1G). The details of the seven hub genes are presented in Table 1.

**Table 1:** Descriptions of the seven hub genes

Gene	Full name	Protein family	Main function	Reference
*GPR4*	G-protein-coupled receptors (GPR) 4	GPCRs family	sensitive to both protons and lysolipids, expressed in multiple neuronal populations and vascular endothelial cells	[38–41]
*SOCS3*	suppressor of cytokine signaling (SOC) 3	SOCS family	positively and negatively regulate macrophage and dendritic cell activation and are involved in T-cell development/differentiation	[42]
*BAG3*	B cell lymphoma-2-associated athanogene (BCL-2-associated athanogene) 3	BAG family	development of both the neuronal and glial components of the central nervous system	[43, 44]
*ZFP36*	Zinc finger protein (ZFP) 36	ZFP family	encoding human tristetraprolin, which regulates TNF-α production by destabilizing TNF-α mRNA	[45, 46]
*CDKN1A*	cyclin-dependent kinase inhibitor p 21 (CDKN1A)	CDKN1 inhibitor family	negative regulation of the cell cycle	[47–49]
*ADAMTS9*	a disintegrin and metalloproteinase with thrombospondin motifs (ADAMTS) 9	ADAMTS protein family	involved in neuroinflammation of the lesion core and neuroplasticity of surrounding tissues	[50, 51]
*GADD45B*	Growth arrest and DNA-damage-inducible protein (*GADD*) 45 beta	*GADD45* family	anti-apoptotic factor in nonneuronal cells, an intrinsic neuroprotective molecule in neurons	[52]

### Expression levels of the seven hub genes

**Fig. 2:**
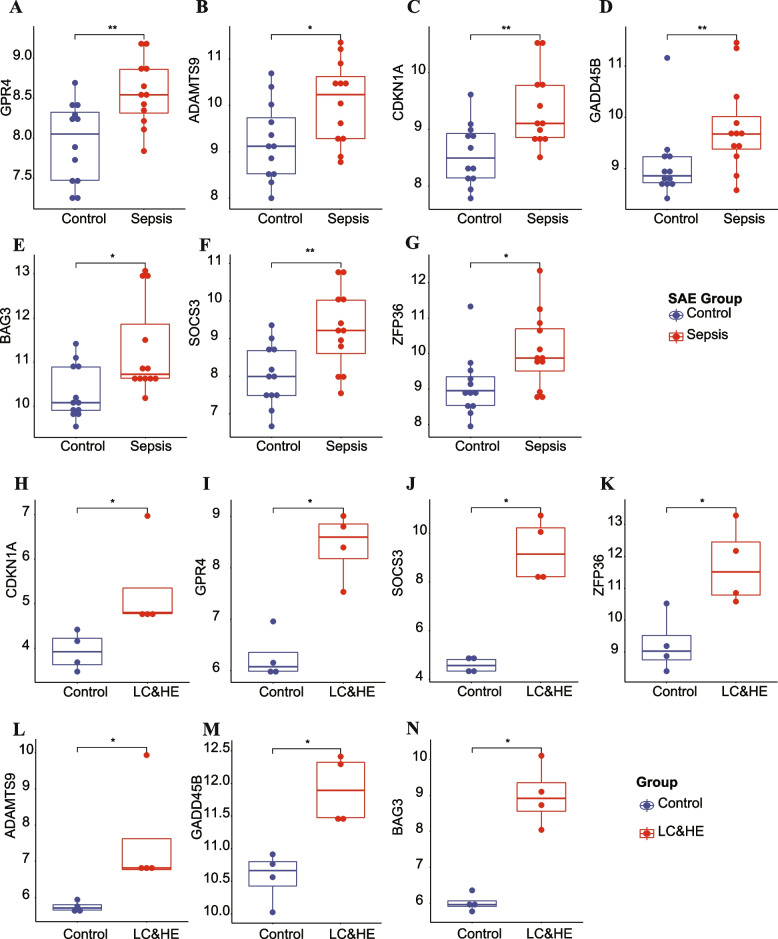
All seven hub genes were significantly highly expressed in SAE/HE compared with that in controls. **A**-**G** The seven hub genes in SAE. **H**-**N** The seven hub genes in HE. SAE, sepsis-associated encephalopathy; LC, liver cirrhosis; HE, hepatic encephalopathy. *P <0.05, **P <0.01

The expression levels of seven hub genes were significantly higher in SAE (Fig. 2A-G) or HE (Fig. 2H-N) than in control group.

### Functional enrichment analysis and construction of protein-protein interaction (PPI) network

**Fig. 3:**
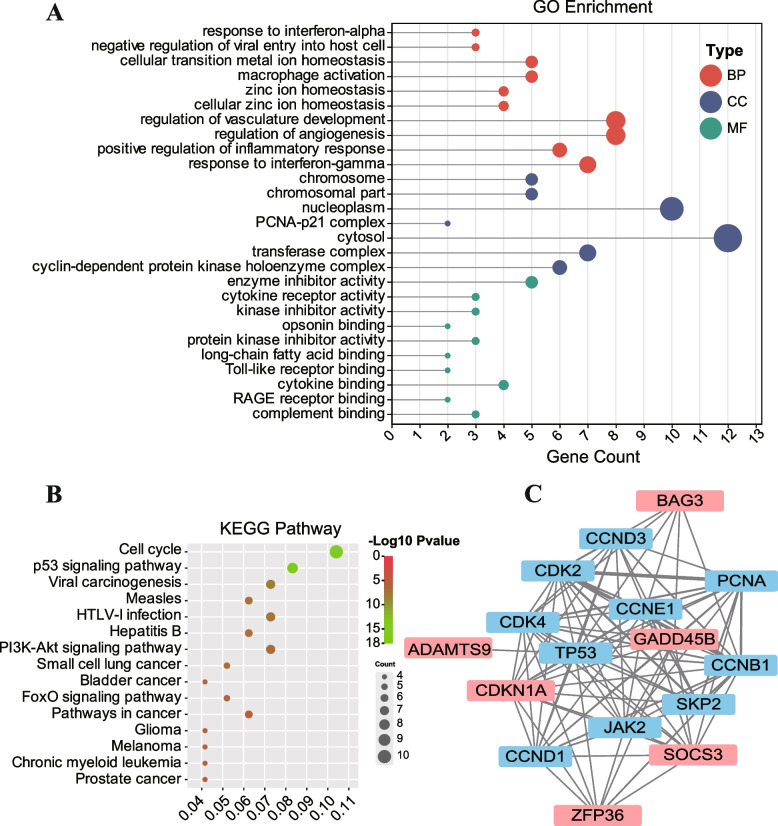
Function enrichment analysis and PPI network of the seven hub genes. **A** GO annotation. BP denotes the biological process; CC indicates the cellular component; MF represents the molecular function. **B** KEGG pathway enrichment analysis. **C** PPI network. The thickness of the lines represents the strength of interaction relationship between each hub gene

We performed GO and KEGG enrichment analyses [33–35] to explore the potential function of the seven hub genes (Fig. 3A, B). GO enrichment analysis comprises three parts: biological process (BP), cellular component (CC), and molecular function (MF) [53]. The BP indicated that these genes were associated with regulation of vasculature development, regulation of angiogenesis, response to interferon-gamma, positive regulation of inflammatory response, cellular transition, metal ion homeostasis, macrophage activation, zinc ion homeostasis, and cellular zinc ion homeostasis (Fig. 3A). Concerning the CC, these genes were involved in cytosol, nucleoplasm, transferase complex, cyclin-dependent protein kinase holoenzyme complex, chromosome, and PCNA-p21 complex (Fig. 3A). In terms of the MF, these seven hub genes were related with enzyme inhibitor activity, cytokine binding, cytokine receptor activity, kinase inhibitor activity, protein kinase inhibitor activity, complement binding, opsonin binding, long-chain fatty acid binding, Toll-like receptor binding, and RAGE receptor binding (Fig. 3A). The KEGG pathway analysis included the cell cycle, the p53 signaling pathway, the PI3K-Akt signaling pathway, and the FoxO signaling pathway (Fig. 3B). The PPI network results revealed that these seven hub genes interacted with each other (Fig. 3C). The detailed interaction relationship of PPI network are presented in Additional file 2: Table S2, which describes the weight of interaction relationship between all genes.

### ROC curves analysis of the seven hub genes

**Fig. 4:**
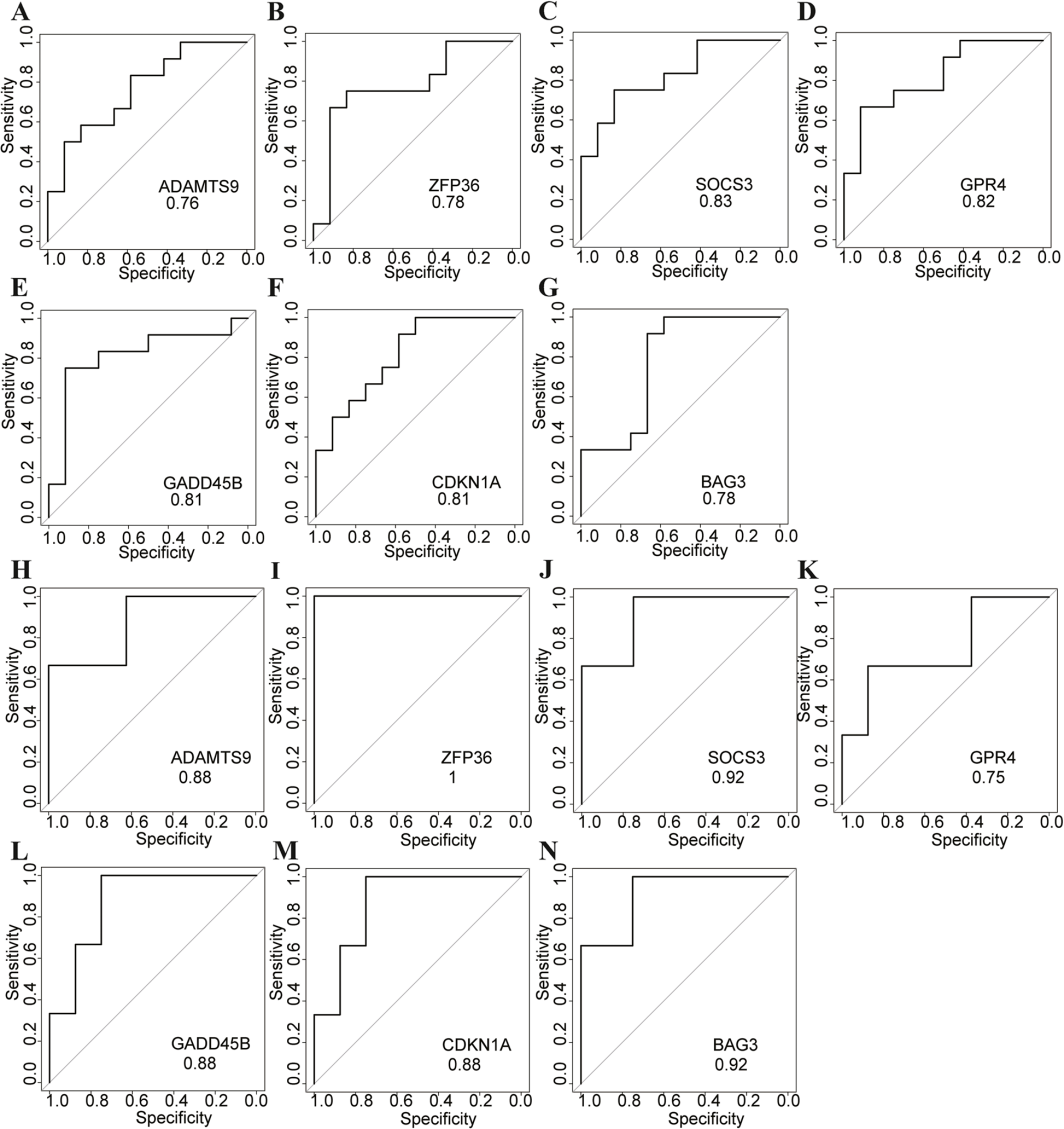
ROC diagnostic efficacy of the seven hub genes in SAE and/or HE. **A**-**G** The seven hub genes in SAE. **H**-**N** The seven hub genes in HE

The AUCs of the seven hub genes, *ADAMTS9*, *ZFP36*, *SOCS3*, *GPR4*, *GADD45B*, *CDKN1A*, and *BAG3*, were 0.76, 0.78, 0.83, 0.82, 0.81, 0.81, and 0.78, respectively, in SAE (Fig. 4A-G) and were 0.88, 1, 0.92, 0.75, 0.88, 0.88, and 0.92, respectively, in HE (Fig. 4H-N). These figures show that the AUCs of these seven hub genes are all greater than 0.7, indicating that these genes have preferable specificity, sensitivity, and excellent diagnostic properties in SAE and/or HE.

### The correlation of the seven hub genes

**Fig. 5:**
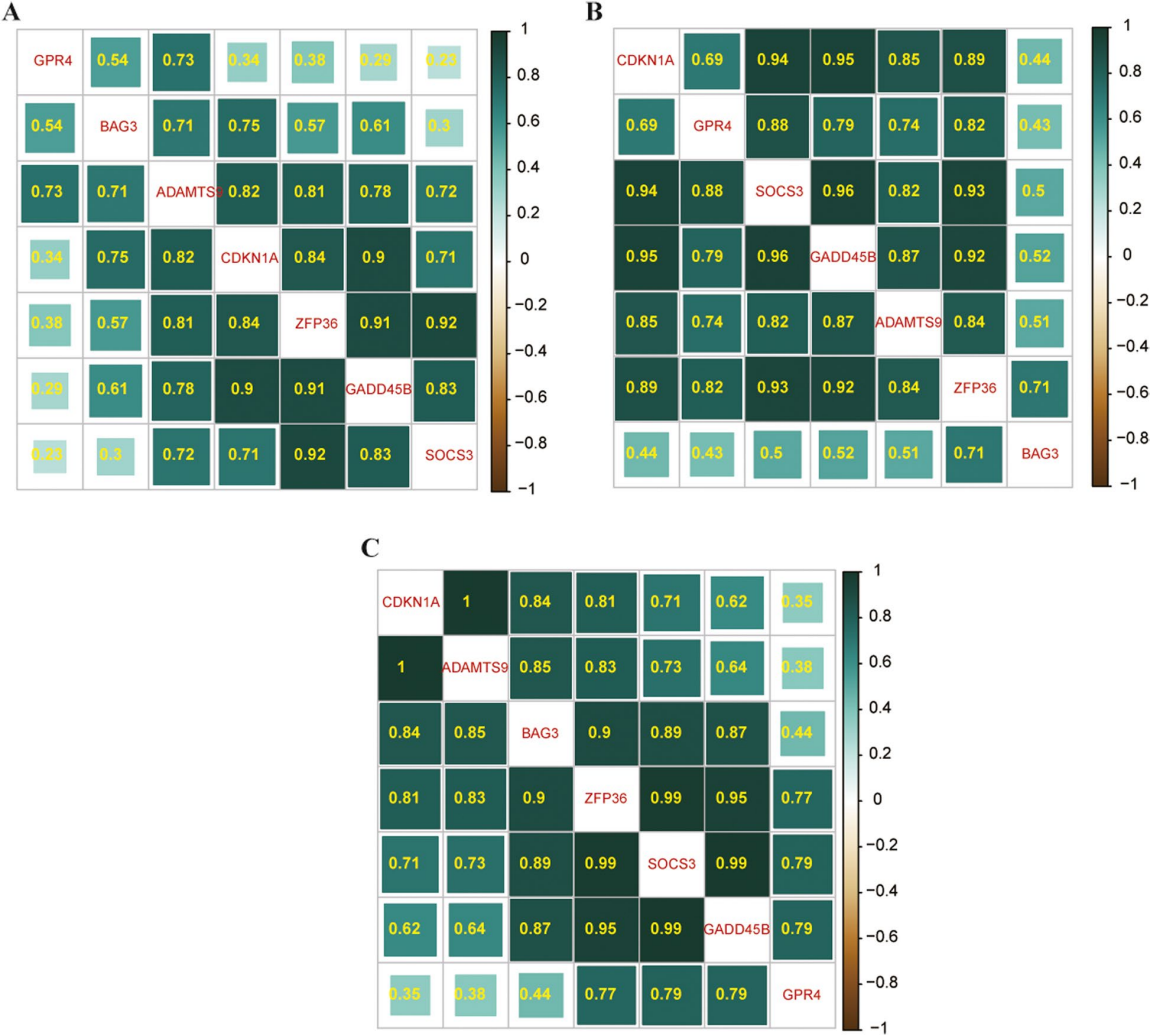
The heatmap of Spearman correlation analysis with the hub genes. **A**-**C** The dataset of GSE135838, GSE57193, and GSE41919, respectively. The greener the color, the greater the correlation coefficient

Figure 5 shows the relationship between the seven DEGs. Figure 5A, B, and C represent three datasets, GSE135838, GSE57193, and GSE41919, respectively; the green color deeper, the stronger the positive correlation between each gene.

### The GESA of the DEGs pathway

**Fig. 6:**
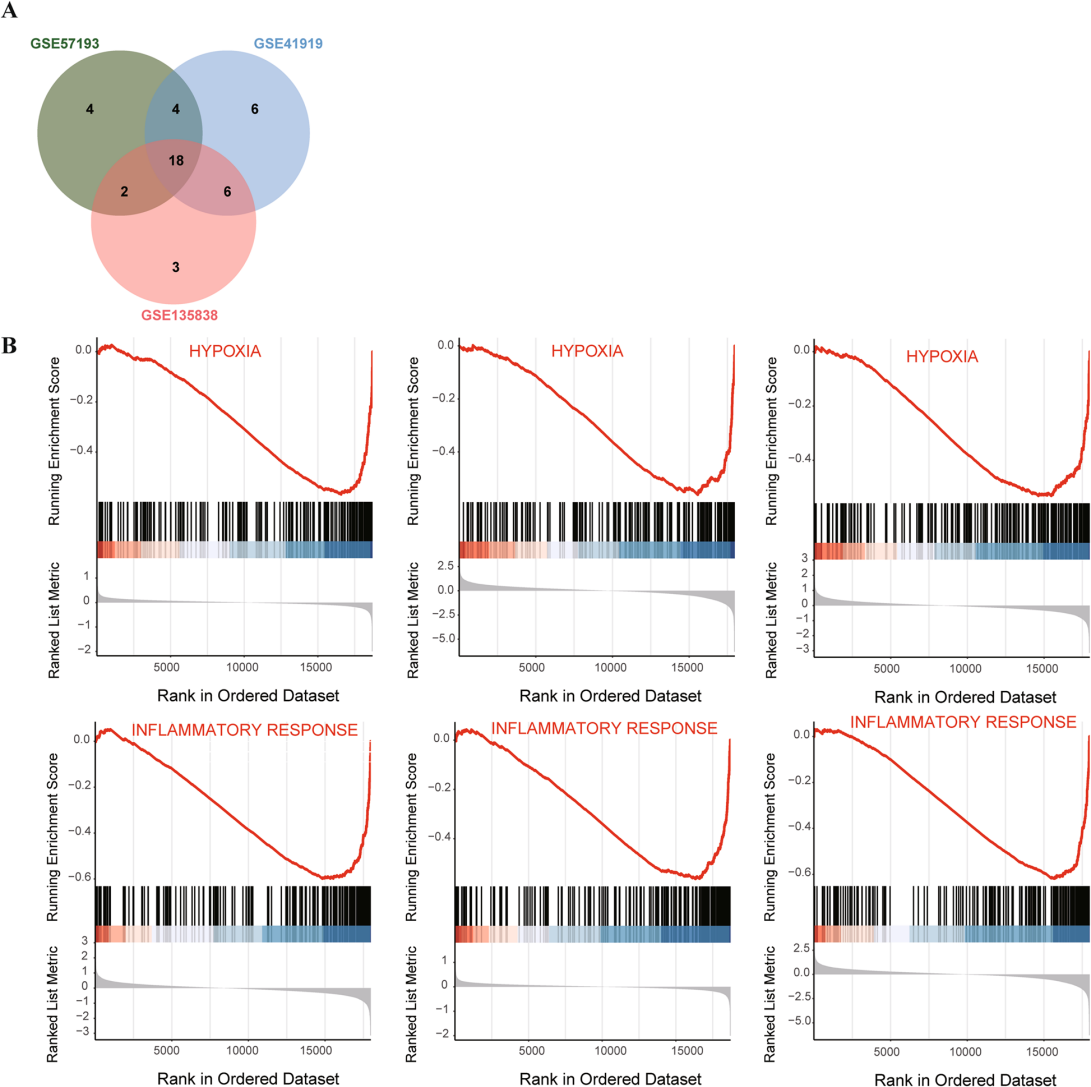
Venn diagram of GSEA results related to three datasets and the GSEA plot. **A** The intersection of signaling pathways. **B** Pathways mainly enriched in hypoxia and inflammatory response

The Venn diagram depicts the intersection of 18 common pathways from the three datasets (Fig. 6A), of which GSEA was mainly enriched in hypoxia and inflammatory response (Fig. 6B). The detailed pathways for the three datasets are shown in Additional file 3: Table S3.

### Immune infiltration analysis

**Fig. 7:**
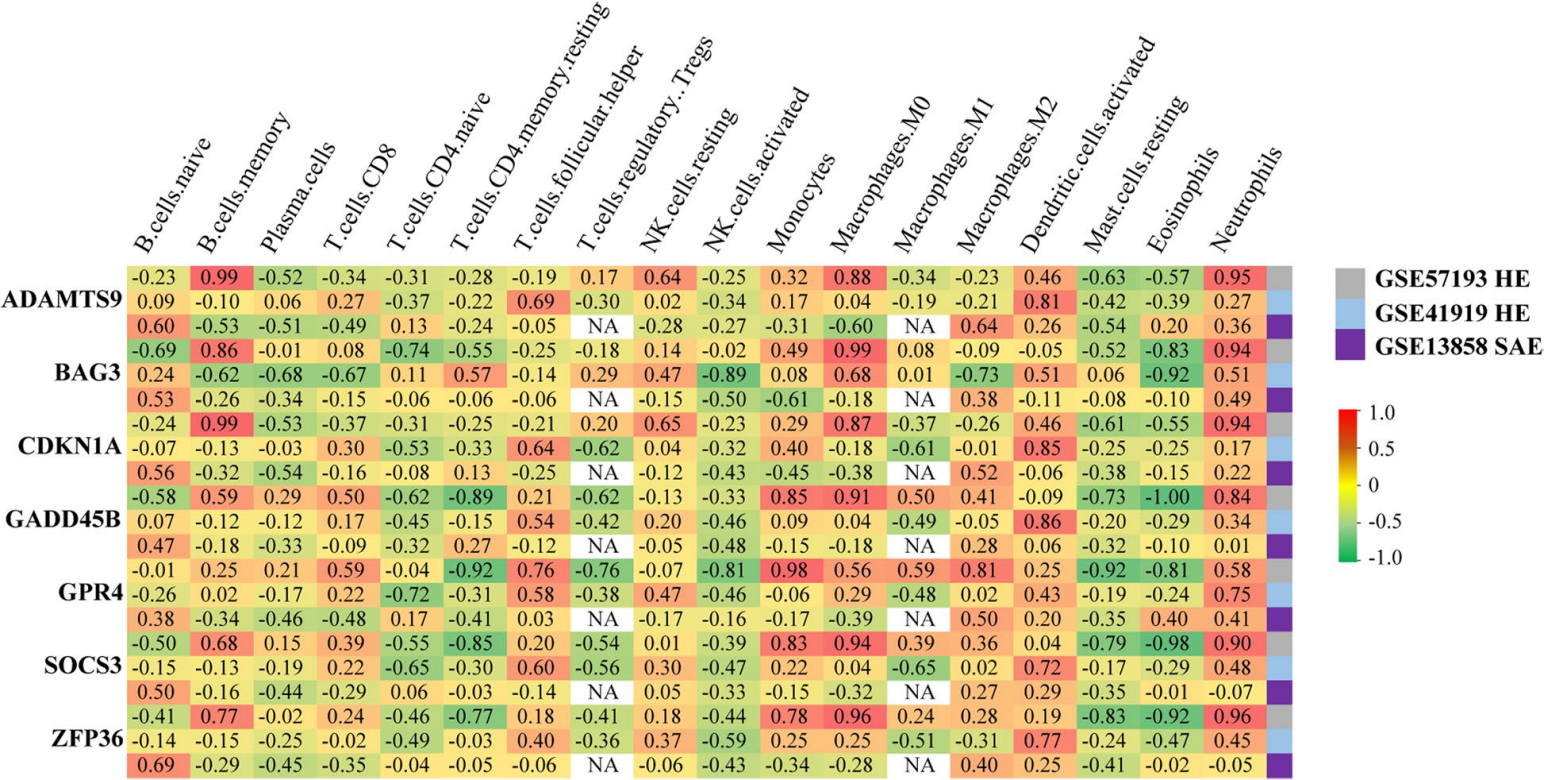
Heatmap of the association between the seven hub genes and immune cell subtype distribution. Red represents a positive correlation, whereas green a negative correlation

CIBERSORT is an analytical tool for immune cell infiltration estimation analysis. Most of the seven genes are closely positively related to innate immune response, including neutrophils, dendritic cells, and macrophage, as well as negatively related with mast cells and NK cells, meanwhile, and also closely related to adaptive immune response comprising B cells and T cells, suggesting that they may be involved in the process of inflammatory and immunization response (Fig. 7).

## Discussion

Cirrhotic patients are at an increased risk of developing sepsis and sepsis-related death and are predisposed to suffer critical complications requiring ICU admission [4, 54]. Septic cirrhotic patients have a significantly increased risk of short- and long-term mortality during their ICU hospital stay [55, 56]. Bacterial infection can act as a triggering factor development of HE [57]. Meanwhile, these extremely critical septic cirrhotic patients have a high risk of developing SAE. Subsequently, HE and SAE are common neurological complications that can co-occur and are difficult to differential diagnose in such patients.

Sepsis 3.0 is a life-threatening organ dysfunction that caused by a dysregulated host response to infection [58]. With an in-depth understanding of critical care medicine, studies have found that although critically ill patients are the most heterogeneous group in ICU, different critical illnesses often exhibit a similar clinical trajectory, in which the response to various acute critical conditions is relatively homogenous and may develop a critical illness [59]. PIRO model (predisposition, insult, response, organ dysfunction) is a well staging system for acute illness, which indicates the heterogeneity of critically ill patients and the identity of clinical manifestations that result from the body’s baseline physiology, the nature of the insult, and the way organ systems respond [60, 61]. Hence, the body’s response disorder usually induces hemodynamic disorders, hypoxia, and subsequent impairment of microcirculation, and mitochondria, resulting in lethal MODS, such as heart or brain, which may endanger the patients’ lives [62–64].

Cirrhotic patients are susceptible to bacterial infection insults, which share similar hemodynamic features to septic shock patients [65, 66]. The infection act as the “first hit”, triggering further dysregulated host response, and then the continuous dysregulated host response leads to a “second hit” to the body, causing MODS, such as SAE and/or HE [59, 67].

Therefore, we chose RNA-seq data from extremely precious autopsy brain tissue of HE and SAE deprived of GEO datasets to explore the possible underlying mutual genes, which maybe the molecular basis of mutual host-organ unregulated response [59] of the two kinds of encephalopathy, to provide new clues and perspectives for the diagnosis and treatment of the two common types of encephalopathy in such septic cirrhotic patients.

Based on the bioinformatics approach, we obtained seven hub genes: *GPR4*, *SOCS3*, *BAG3*, *ZFP36*, *CDKN1A*, *ADAMTS9*, and *GADD45B*. The expression levels of those genes were higher than that in the control group, and their AUCs were greater than 0.7, suggesting that their excellent diagnostic efficacy and performance in SAE and/or HE. As shown in Fig. 3, the GO and KEGG enrichment analysis suggested that these genes could be related to important biological processes, such as regulation of vasculature development and angiogenesis, cell cycle, cytokine receptor activity, macrophage activation, positive regulation of inflammatory response, and are associated with the p53 signaling pathway, PI3K-Akt signaling pathway, and FoxO signaling pathway. The detailed descriptions about the seven hub genes are shown in Table 1. *CDKN1A*—a member of the cyclin-dependent kinase inhibitor protein family—plays critical roles in the negative regulation of the cell cycle [47–49]. Sepsis, often complicated with hypoxia, which leads to cell cycle arrest, is linked to the transcription of CDKN1A [68]. Whereas hypoxia is usually accompanied by acidosis, in SAE or HE. There was a good linear relationship between cerebral blood flow and PaCO_2_, in which acidosis and hyperventilation may easily lead to brain ischemia [22]. *GPR4* is a subfamily of G-protein-coupled receptors sensitive to both protons and lysolipids [38, 39]; hence, a *GPR4* blocker—NE52-QQ57—can reduces CO_2_-induced hyperventilation that maybe the potential therapeutic target. *GADD45B* is an anti-apoptotic factor in nonneuronal cells and is an intrinsic neuroprotective molecule in neurons; its expression is up-regulated following focal and global cerebral ischemia, and it has a direct beneficial effect in cerebral ischemic injury [52, 69].

Furthermore, GSEA and immune infiltration analysis suggested that these genes could be involved in mutually intricate processes, such as hypoxia, inflammatory, and immunoreaction responses. As shown in Fig. 7, the seven hub genes are closely positively related to innate immune response, including neutrophils, dendritic cells, monocytes, and macrophages M0/M2, as well as negatively related to mast cells and activated NK cells.

After pathogenic microorganisms invade the human body, that is, after the body accepts the “first hit”, the host’s innate immune system is activated, comprising monocytes/macrophages, NK cells, neutrophils, dendritic cells, and other innate immune cells [70]. Infiltrating monocytes/macrophages and activation of microglia are closely related to excessive neuroinflammation in SAE [71]. Microglia is a type of macrophage distributed in all parts of the brain and critical immune cells in the nervous system, and microglia can be activated by inflammatory factors to release further proinflammatory cytokines [72], which is a considerable pathogenesis of SAE and/or HE [73, 74] and may be closely related to PI3K/AKT signaling pathway and FoxO signaling pathway [75–79] (Fig. 3).

In most cases, the host’s innate immune response can eliminate the invading pathogen. However, sometimes the host response gets overwhelmed and becomes further unbalanced and harmful [80], known as the “second hit”. Once the body has acquired a “second hit” and developed a critical sepsis illness, the central nervous system plays a vital role in responding to the “hit” and maintaining homeostasis. Subsequently, the body exhibits a series of stress-related decompensation syndrome, including the inflammatory immune system, vascular endothelial system, coagulation system, metabolism, and bioenergy. The inflammatory and immunoreaction responses may share the common downstream pathways, which are mainly characterized by the activation of several inflammatory cells and the release of proinflammatory mediators in the early stage; then, with the release of inflammatory mediators and immune cell apoptosis, the body enters a state of significant immunosuppression in both the innate and adaptive immune systems [59, 81–83], such as apoptosis of B cells and the decrease of CD4^+^/CD8^+^ T cell ratio [80, 84]. As shown in Fig. 7, the seven hub genes closely related to adaptive immune response were negatively correlated with plasma cells and CD4^+^ T cells and positively correlated with CD8^+^ T cells. Among them, *SOCS3* can positively and negatively regulate macrophage and dendritic cell activation and are essential for T-cell development and differentiation [42], which may be involved in the highly complex neuroimmune response. To sum up, inflammation and the immune process interact closely, which is the underlying pathophysiological mechanism of the host response. The aforementioned seven hub genes are closely related and may be the mutual molecular mechanism of the host-organ dysregulated response in brain tissue.

The evolution of critical care medicine had two stages. The first stage is characterized by maintaining homeostasis and focusing on pathophysiology at the organ-level. In the current second stage, the main feature is transformed into a deeper understanding of the pathophysiological mechanism of the host response derived from various common ICU syndromes because of the complexity and heterogeneity of critical illness. Hence, identifying critical illness as a stress-related host response decompensation may assist us in improving current critical management [83]. However, excellent diagnostic biomarkers and molecular targets of host-organ dysregulated response for SAE and/or HE are unavailable in septic cirrhotic patients. The seven hub genes exhibit excellent diagnostic efficacy and have the potential to become promising central therapeutic targets blocked in host-organ dysregulated response, providing underlying molecular mechanisms for precise clinical treatment of such septic cirrhotic patients; earlier recognition and timely intervention are crucial for ICU management of such septic cirrhotic patients. Nevertheless, in the present study, the sample size was small because of the rarity and difficulty of obtaining autopsy brain tissue; therefore, we rely heavily on public autopsy brain sample data for analysis, with relevant conclusions and explicit molecular mechanisms to be experimentally verified in future work.

## Conclusion

In summary, we identified the seven hub genes as potential diagnostic biomarkers and therapeutic targets in septic cirrhotic patients with HE and/or SAE. Hypoxia, inflammatory, and immunoreaction responses may share the common downstream pathways of the two common encephalopathies, SAE and HE, for which earlier recognition and timely intervention are crucial for management of such septic cirrhotic patients in ICU.

### Supplementary Information

**Additional file 1:** **Table S1.** The genes list of Green and Turquoise modules.

**Additional file 2:** **Table S2. **The detailed interaction relationship of PPI network.

**Additional file 3:** **Table S3.** The detailed pathways deprived from the three datasets.

## Data Availability

The data and materials in the current study are available from the corresponding author upon reasonable request. All raw and processed data are freely available from GEO database (https://www.ncbi.nlm.nih.gov/geo/).

## Abbreviations

ICUs: Intensive Care Units
SAE: Sepsis-associated encephalopathy
HE: Hepatic encephalopathy
MODS: Multiple Organ Dysfunction Syndrome
CBF/CMRO_2_: Cerebral blood flow/cerebral metabolic rate of oxygen consumption
GEO: Gene Expression Omnibus
DEGs: Differentially expressed genes
WGCNA: Weighted gene co-expression network analysis
GSEA: Gene Set Enrichment Analysis
GO: Gene Ontology enrichment terms
KEGG: Kyoto Encyclopedia of Genes and Genomes
BP: Biological process
CC: Cellular component
MF: Molecular function
PPI: Protein-protein interaction
